# The First Iranian Patient with West Syndrome Due to a *RYR3* Gene Variant: A Case Report and Literature Review

**DOI:** 10.34172/aim.35157

**Published:** 2026-01-01

**Authors:** Behzad Haj Mohammad Hassani, Niloofar Ghasemi, Kianoosh Malekzadeh

**Affiliations:** ^1^Department of Medical Genetics, Faculty of Medicine, Hormozgan University of Medical Sciences, Bandar Abbas, Iran; ^2^Molecular Medicine Research Center, Hormozgan Health Institute, Hormozgan University of Medical Sciences, Bandar Abbas, Iran

**Keywords:** Calcium release channel, Infantile spasm, Ryanodine receptor 3, *RYR3*, Syndrome, West syndrome

## Abstract

West syndrome (WS) is a severe developmental and epileptic encephalopathy of infancy, defined by the triad of epileptic spasms, hypsarrhythmic electroencephalography, and developmental delay. Recent limited studies have suggested that the *RYR3* gene is involved in the pathogenesis of WS. Our study presents an Iranian patient with WS and aims to provide further evidence to support the role of *RYR3* variants in this condition. The proband was a male patient aged 2 years and 2 months with drug-resistant epileptic spasms and developmental regression. Trio-based whole-exome sequencing (Trio-WES) was conducted, and a novel *de novo* heterozygous variant in *RYR3* (NM_001036.6: c.4112T>C, p.Leu1371Pro) was identified in the proband. The variant was not found in population databases, and bioinformatics analyses also predicted the deleterious impact of the variant. This study describes a novel *RYR3* variant in a patient with WS, supporting a potential pathogenic role for *RYR3* in early-onset epileptic encephalopathy. Therefore, it should be considered during the genetic assessment of unexplained WS cases.

## Introduction

 West syndrome (WS), also referred to as infantile spasms, is a rare brain disorder of infancy. It is characterized by epileptic spasms, a specific pattern in electroencephalography (EEG) called hypsarrhythmia, and developmental regression. WS can have long-term consequences and many children develop drug-resistant epilepsy, learning disability, and traits of autism. The causes of WS are varied and include genetic, structural, and metabolic reasons. However, the precise cause is frequently unidentified despite thorough assessment.^1^

 Next-generation sequencing has increasingly identified both *de novo* and inherited variants in genes involved in brain development, synaptic function, and calcium (Ca^2+^) homeostasis as key contributors to this syndrome. Among the genes recently implicated in epilepsy syndromes is *RYR3*, which encodes ryanodine receptor 3.^2^ In addition to RYR1 and RYR2 proteins, RYR3 also regulates intracellular calcium signaling. This regulation plays a crucial role in different cellular functions, including muscle contraction, synaptic neurotransmitter release, and neuronal excitability. While RYR1 and RYR2 are expressed in the skeletal and heart muscle, respectively, RYR3 is highly expressed in the central nervous system (CNS), particularly in the cerebral cortex, cerebellum, and hippocampus.^3^

 Calcium signaling plays an important role in achieving and maintaining a normally functioning CNS in mammals. Disruption of intracellular calcium dynamics can negatively impact synaptic plasticity, neuron firing patterns, and gene transcription. All of these factors contribute to typical motor and cognitive development.^4^ Initial research using mouse knockout models demonstrated that the total absence of RYR3 results in behavioral and electrophysiological deficits, including a lack of long-term potentiation (LTP) and poor learning of spatial tasks. Therefore, confirming its involvement in CNS neuronal Ca^2+^ homeostasis. Additionally, the sexual and survival phenotypes of RYR3-null mice indicate that its major role is specifically in the CNS.^5^ Thus, calcium channel function in the development of epilepsy is established, and calcium homeostasis-regulating gene mutations have been identified for a few of the epilepsy syndromes, including early infantile epileptic encephalopathies.^6^

 To date, only four *RYR3* gene variants have been identified or characterized in relation to WS. This study reports a novel *RYR3* gene variant in a child with WS, providing further evidence that supports the potential role of *RYR3* as a candidate gene in the pathogenesis of the syndrome.

## Case Report

 Our subject was a boy aged 2 years and 2 months with developmental delay and drug-resistant seizures. He was the third child of non-consanguineous Iranian parents, born at 38 weeks without any complications, weighing 3.2 kg at birth. The family members had no history of seizures or neurological conditions.

 The patient’s early development was normal until approximately 4 months of age, when he began experiencing clusters of sudden flexor-extensor epileptic spasms involving the neck, trunk, and limbs, which mostly occurred during transitional states between wakefulness and sleep, and after feeding. Initially, the seizures were sporadic but rapidly progressed to multiple clusters each day of 50‒90 spasms per cluster. Initial EEG demonstrated a hypsarrhythmic pattern with multifocal spikes and a chaotic background. Brain MRI showed no cortical dysplasia or structural damage. Ammonia and lactate levels, plasma amino acid analysis, urine organic acid screening, and acylcarnitine profile were in the normal ranges.

 The patient, diagnosed with West syndrome, was first given ACTH along with valproic acid and clobazam. The spasms lessened initially, but the seizures returned after four days. In the next few months, prednisolone, valproic acid, and clonazepam were tried, and only some improvement was observed.

 At 12 months of age, the previously acquired milestones he had achieved (such as pulling to stand, responding to his name, and babbling) began to regress. Based on examination, he could only sit briefly with assistance, did not make meaningful sounds, and showed limited eye contact and poor social skills. During the last follow-up (at 2 years and 2 months of age), the patient remained on a combination of valproic acid, topiramate, clonazepam, and a modified ketogenic diet. He continued to experience more than four daily seizure clusters, each consisting of 20–40 spasms. His development remained severely delayed, with no speech, unable to walk independently, and interacting poorly with caregivers.

 Genomic DNA was extracted from the proband’s peripheral blood samples and both biological parents using standard protocols. Trio-whole exome sequencing (Trio-WES) was conducted to detect potentially disease-causing variants related to the proband’s phenotype. The SureSelectQXT Reagent Kit (Agilent Technologies, Santa Clara, CA) was prepared for library preparation. Paired-end sequencing was performed at 100X coverage on a NovaSeq6000 (Illumina, San Diego, CA, USA). The variants were filtered based on minor allele frequency (MAF) and guidelines from the American College of Medical Genetics and Genomics (ACMG) (Table S1). Consequently, Sanger sequencing was used to confirm the variants in the proband.

**Figure 1:**
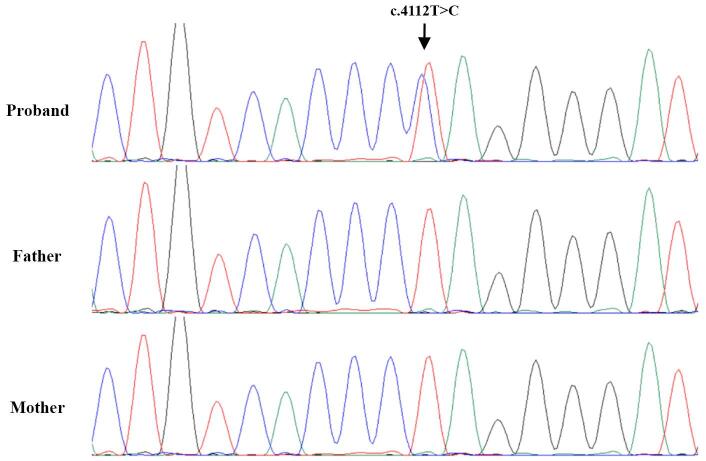
Sanger sequencing of the *RYR3* variant. The proband was identified as a heterozygote for the c.4112T > C variant, whereas his parents exhibited a wild-type genotype.

**Figure 2:**
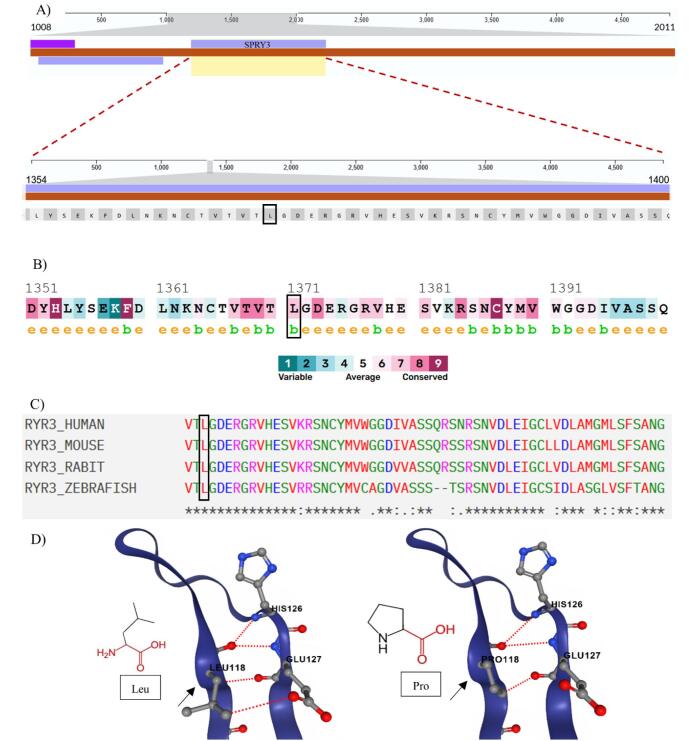
Amino acid position and conservation. **A)** Leu 1371 is located in the SPRY3 domain of the RYR3 protein, which is highly conserved based on **B)** ConSurf and **C)** Clustal Omega. **D)** Substitution of Leu with Pro disrupts local interactions and eliminates a hydrogen bond. Residues are displayed according to their positions within the SPRY3 domain.

 WES detected a novel heterozygous variant in the *RYR3* gene (NM_001036.6: c.4112T > C, p.Leu1371Pro) in the proband, which was not present in either parent. Sanger sequencing was subsequently performed and confirmed the presence of the variant (Figure 1). The variant was not observed in gnomAD (https://gnomad.broadinstitute.org) and the Iranome databases (https://www.iranome.com) (a population-specific resource containing exome data from 1,200 unrelated Iranian individuals of diverse ethnicities).^7^ According to the ACMG guidelines, this variant met the PM1, PM2, and PP3 criteria. Analyses using various *in-silico* tools, including HOPE, DynaMut2, and Mutation Taster, predicted the deleterious effect of the variant on the conserved SPRY3 domain of *RYR3* (Table 1). Conservation assessment with ConSurf and Clustal Omega revealed that the substituted residue is highly conserved across species (Figure 2A-C), supporting a functional impact for this variant. The effect of this substitution on conformation is also evident in Figure 2D.

**Table 1:** *In-silico *predictions of the c.4112T>C variant.

***In-silico*** ** tool**	**Prediction**	**Score**
MetaRNN	Pathogenic	0.93
REVEL	Pathogenic	0.68
Polyphen-2	Deleterious	0.979
CADD	pathogenic	26.5
MutPred	Pathogenic	0.875
DANN	Pathogenic	0.984
DEOGEN2	Deleterious	0.712
AlphaMissense	Pathogenic	0.995
ClinPred	Pathogenic	0.997
PROVEN	Deleterious	-5.14
FATHMM-XF	Deleterious	0.962
Mutation assessor	Pathogenic	2.6
SIFT	Pathogenic	0.02
Mutation Taster	Deleterious	0.8
GERP	High Constraint	5.1

## Discussion

 WS is a severe developmental epileptic encephalopathy characterized by early onset epileptic spasms, a special EEG pattern (hypsarrhythmia), and progressive cognitive and motor impairment.^1^ Although several genes have been implicated in WS, recent evidence suggests that *RYR3* may represent a novel candidate gene involved in the pathogenesis of this condition.^1,2^ The *RYR3* gene is located on the long arm of chromosome 15 and has 34 transcripts, the principal transcript encoding a protein of 4870 amino acids. This protein is a type of ryanodine receptor, which functions as a calcium release channel on the endoplasmic reticulum, mediating the efflux of calcium from intracellular stores.

 The spatial expression pattern suggests that RYR3 is a central modulator of synaptic calcium signaling and neuronal plasticity, processes that are often disrupted in epileptic encephalopathies. Clinical observations indicate that some individuals with *RYR3*-related epilepsy show limited or no response to first-line treatments, including vigabatrin or corticosteroids, further highlighting the potential contribution of calcium channel dysfunction in drug-resistant forms of infantile epilepsy.^2,6^

 The present study identified a novel *RYR3* variant (c.4112T > C) in a patient with West syndrome. The absence of variants in population databases, combined with supportive *in-silico* predictions, showed its pathogenicity (Table 1). GERP, ConSurf, and Clustal Omega analyses revealed a high degree of conservation at position Leu1371, which is located in the conserved domain of SPRY3 (the 118^th^ amino acid of the domain). Furthermore, considering the distinctive conformational constraints of proline, the leucine-to-proline substitution is likely to be detrimental to the protein’s function. Collectively, these findings support the deleterious impact of the variant on protein structure and function.

 The genotype-phenotype correlation involving *RYR3* variants appears to be complex. Initially, the loss-of-function variants of this gene were reported with congenital myopathy. Conversely, four previous prominent case and cohort studies have linked gain-of-function *RYR3* variants to epilepsy phenotypes ranging from generalized seizures to developmental and epileptic encephalopathies (DEEs).

 The first report by Appenzeller *et al.* described two patients with likely pathogenic *RYR3* variants. The first patient carried an in-frame deletion (c.9603-9605delTCA, p.Ile3202del) and was diagnosed with Lennox–Gastaut syndrome. He experienced multiple daily seizures, including head bobbing, atonic and tonic seizures, and developmental delay. The second patient harbored a missense variant (c.14104G > A, p.Asp4702Asn) and was diagnosed with epileptic encephalopathy, exhibiting infantile spasms at 6 months and achieving seizure freedom by 11 months of age. Notably, both patients showed multifocal or hypsarrhythmic EEG patterns, with no evidence of congenital myopathy or overt structural brain anomalies, thereby supporting a central neurological role for RYR3 dysfunction.^8^

 In the second study, Peng *et al.* identified additional missense *RYR3* variants (c.3716A > G, p.Lys1239Arg and c.4046C > T, p.Thr1349Ile) in two children with WS. While these variants were categorized as variants of uncertain significance (VUS), their co-occurrence with early onset epileptic spasms and global developmental delay supported the hypothesis that *RYR3* is a candidate gene for infantile epilepsies. However, the absence of functional validation or inheritance data limited definitive conclusions regarding causality.^2^

 The third study by Li *et al.* reported the c.10943C > T (p.Thr3648Met) *de novo* variant in a patient with DEE and West syndrome phenotype. This variant led to early-onset spasms beginning at 8 months of age, severe developmental regression, drug-resistant epilepsy, and characteristic EEG findings of multifocal discharges and hypsarrhythmia. The patient had no structural brain abnormalities on MRI, emphasizing the likely molecular (rather than anatomical) basis of the disorder. Functional predictions and the low frequency of this variant from population databases further supported its pathogenicity.^9^

 A more recent study by Tian *et al.* extended the phenotypic spectrum by reporting seven unrelated individuals with non-lesional focal epilepsy who harbored either compound heterozygous or *de novo *missense variants in *RYR3*. Although the clinical presentations were milder than classical DEE, mainly focal seizures with or without febrile seizures, the authors proposed a model of genetic dependence nature, wherein partial loss or gain of function in *RYR3* may not be sufficient to cause disease alone but can increase susceptibility to epileptic manifestations. All affected individuals were seizure-free at follow-up, and neurodevelopment was mostly preserved, suggesting that these variants act as genetic modifiers rather than direct causes.^10^

 Notably, two rare variants affecting conserved residues within the SPRY3 domain, including a reported variant by Peng *et al. *and the novel (c.4112T > C, p.Leu1371Pro) variant identified in our study, highlight the potential critical role of this domain in maintaining calcium signaling fidelity during early brain development. Dysregulated calcium homeostasis via dysfunctional RYR3 channels may promote epileptogenicity by disrupting synaptic signaling, increasing network excitability, or impairing the function of inhibitory interneurons.^4,8^

 Despite emerging evidence, *RYR3* has not yet been incorporated into most standard epilepsy gene panels. However, the identification of pathogenic variants through trio-based sequencing approaches, including in patients with no prior structural or metabolic abnormalities, supports the need to expand the molecular screening of infants with unexplained spasms to include this gene. This approach may aid in early diagnosis, inform management decisions, and prompt research into targeted therapeutics that aim to modulate intracellular calcium dynamics.

 Further functional investigations are required to elucidate the pathogenesis of *RYR3* variants. Additional reports of variants in this gene in patients with WS and other types of epilepsy will provide new insights. Ongoing collection of the genotype–phenotype data from diverse populations will further improve our understanding of the disease spectrum and could identify regions within *RYR3*, like SPRY3, as possible targets for therapy.

## Conclusion

 In conclusion, this study reports a novel heterozygous missense variant (c.4112T > C) in the *RYR3* gene in a patient with West syndrome and expanding the mutational and phenotypic spectrum associated with this calcium channel gene. Combined with previous reports of *RYR3* variants in DEEs, these findings underscore the gene’s role in severe early-onset epilepsy.

## Supplementary Files

Supplementary file 1 contains Table S1.
