# Supplementary material for: The First Iranian Patient with West Syndrome Due to a RYR3 Gene Variant: A Case Report and Literature Review
Source: Arch Iran Med. 2026 Jan 1;29(1):53–7. doi: 10.34172/aim.35157 (PMC13338499; doi:10.34172/aim.35157)
Supplement: Supplementary file 1 — contains Table S1. [file aim-29-53-s001.pdf]

Arch Iran Med. January 2026;29(1):53-S1

doi:10.34172/aim.35157

http://journalaim.com

Table S1. List of final filtered variants. Variants were identified after the initial filtering steps based on epilepsy-related gene panels and allele frequency thresholds. Among the filtered variants, fewer than ten were considered potentially associated with the disease and had passed the allele frequency filter. However, except for the *RYR3* variant, the remaining variants were excluded due to various reasons, such as a lack of confirmation by Sanger sequencing (resulting from low WES read depth) or classification as benign/likely benign according to previous studies and *in silico* analyses. VUS, Variant of Uncertain Significance; NF, Not found.

Variant ID	Gene	Transcript	Zygosity (Patient)	Segregation (Trio)	Frequency (gnomAD)	Note
c.4112T>C	RYR3	NM_001036.6	Heterozygous	De novo	NF	Confirmed by Sanger sequencing
c.44T>C	GABRA1	NM_001127644.2	Heterozygous	De novo	NF	VUS; Not confirmed by Sanger sequencing
c.247C>T	CACNA1H	NM_021098.3	Heterozygous	Maternal	NF	VUS; Not confirmed by Sanger sequencing
c.327C>G	CHD2	NM_001271.4	Heterozygous	Maternal	0.00004	Likely Benign variant
c.336C>T	KCNQ3	NM_004519.4	Heterozygous	Paternal	0.000004	Likely Benign variant
c.711G>A	SLC13A5	NM_177550.5	Heterozygous	De novo	NF	VUS; Not confirmed by Sanger sequencing
c.537C>T	CHRNA2	NM_000742.4	Heterozygous	Maternal	0.000024	Likely Benign variant
c.66C>T	PNKP	NM_007254.4	Heterozygous	Paternal	0.00003188	Likely Benign variant
C.164C>T	GOSR2	NM_004287.5	Heterozygous	Maternal	NF	VUS; Not confirmed by Sanger sequencing
